# Evaluating Online Cannabis Health Information for Thai Breast Cancer Survivors Using the Quality Evaluation Scoring Tool (QUEST): Mixed Method Study

**DOI:** 10.2196/55300

**Published:** 2024-12-24

**Authors:** Thanarpan Peerawong, Tharin Phenwan, Meiko Makita, Sojirat Supanichwatana, Panupong Puttarak, Naowanit Siammai, Prakaidao Sunthorn

**Affiliations:** 1 Radiation Oncology Unit, Department of Radiology, Faculty of Medicine, Prince of Songkla University, 15 Kanjanavanich Rd., Songkhla, 90110, Thailand, 66 074451502; 2 School of Health Sciences, University of Dundee, Dundee, United Kingdom; 3 Faculty of Pharmaceutical Sciences, Prince of Songkla University, Songkhla, Thailand; 4 Tanyawej Breast Center, Songklanagarind Hospital, Prince of Songkla University, Songkhla, Thailand; 5 Chom-rom-nom-yen, Breast Cancer Survivors Support Group, Songklanagarind Hospital, Prince of Songkla University, Songkhla, Thailand

**Keywords:** cannabis, medical cannabis, Thailand, critical discourse analysis, mixed method study, breast cancer, digital literacy, legislation, health literacy

## Abstract

**Background:**

Following medical cannabis legalization in Thailand in 2019, more people are seeking medical cannabis–related information, including women living with breast cancer. The extent to which they access cannabis-related information from internet sources and social media platforms and the quality of such content are relatively unknown and need further evaluation.

**Objective:**

This study aims to analyze the factors determining cannabis-related content quality for breast cancer care from internet sources and on social media platforms and examine the characteristics of such content accessed and consumed by Thai breast cancer survivors.

**Methods:**

A mixed methods study was conducted between January 2021 and May 2022, involving a breast cancer survivor support group. The group identified medical cannabis–related content from frequently accessed internet sources and social media platforms. The contents were categorized based on content creators, platforms, content category, and upload dates. Four researchers used the Quality Evaluation Scoring Tool (QUEST) to assess content quality, with scores ranging from 0 to 28. Contents were expert-rated as either high or poor. The QUEST interobserver reliability was analyzed. Receiver-operating characteristic curve analysis with the Youden index was used to determine the QUEST score cut-off point. Statistical significance was set at *P*<.05. Fairclough Critical Discourse Analysis was undertaken to examine the underlying discourses around poor-quality content.

**Results:**

Sixty-two Thai-language cannabis-related items were evaluated. The content sources were categorized as follows: news channels (21/62, 34%), government sources (16/62, 26%), health care providers (12/62, 19%), and alternative medicine providers (12/62, 19%). Most of the contents (30/62, 48%) were uploaded to YouTube, whereas 31% (19/62) appeared on websites and Facebook. Forty of 62 content items (64%) were news-related and generic cannabis advertisements while 8 of 62 (13%) content items had no identifiable date. The interobserver QUEST score correlation was 0.86 (*P*<.001). The mean QUEST score was 12.1 (SD 7.6). Contents were considered “high” when the expert rating was >3. With a QUEST score of 15 as the threshold, the sensitivity and specificity for differentiating between high and poor content quality were 81% and 98%, respectively. Content creation was the only significant factor between high- and poor-quality content. Poor-quality contents were primarily created by alternative medicine providers and news channels. Two discourses were identified: advocacy for cannabis use normalization and cannabis romanticization as a panacea. These discourses overly normalize and romanticize the use of cannabis, focusing on indications and instructions for cannabis use, and medical cannabis promotion, while neglecting discussions on cannabis contraindications and potential side effects.

**Conclusions:**

The varying quality of medical cannabis–related information on internet sources and social media platforms accessed and shared by Thai breast cancer survivors is an issue of concern. Given that content creators are the sole predictive factors of high content quality, future studies should examine a wider range of cannabis-related sources accessible to both the public and patients to gain a more comprehensive understanding of the issue.

## Introduction

Globally, breast cancer is the most common cancer in women, with an age-standardized rate of 46.8 per 100,000 individuals [1]. The number of new cases is expected to rise from 2.3 million in 2020 to 3.2 million by 2040 [2]. In Thailand, about 21,600 new cases were reported in 2022, with a projection of 25,600 by 2040 [2]. Breast cancer treatments often involve a combination of surgery, radiation therapy, chemotherapy, and targeted therapy. These interventions have proven effective, with a trade-off between side effects, including nausea, vomiting, hair loss, fatigue, neuropathy, and decreased immunity [3].

Currently, alternative medicine approaches are gaining attention as potential complementary therapies to alleviate these side effects and improve patient well-being [4,5]. Hence, medical cannabis use, a complementary therapy, has gained traction in mitigating the side effects of breast cancer treatment [5,6].

Medical cannabis refers to the use of *Cannabis sativa* L. or its extracts for medicinal purposes [5]. The main chemical compounds in cannabis are tetrahydrocannabinol and cannabidiol, which demonstrated anticancer properties in a pre-clinical study [7]. Clinically, there are clear medical indications for cannabis use, including the treatment of intractable chronic pain, nausea, and vomiting [5,8-12].

In Thailand, cannabis has been used as a part of traditional medicine for centuries. The most commonly used forms of cannabis in nontraditional medicine involve the oral intake of crude oil extracts, raw plants (flowers, leaves, or whole plants with roots and stems), and topical skin products.

There are 3 categories of cannabis-based products legalized for medicinal purposes in Thailand. The first category is medicinal grade, which refers to substances that meet rigorous standards of purity, potency, and safety, making them suitable for use in medical treatments and pharmaceutical formulations, with three formulae: (1) high tetrahydrocannabinol concentration (13 mg/mL tetrahydrocannabinol), (2) high cannabidiol concentration (100 mg/mL cannabidiol), and (3) the tetrahydrocannabinol-cannabidiol mixture in a 1:1 ratio (27 mg/mL tetrahydrocannabinol and 25 mg/mL cannabidiol) [13]. The second category comprises 16 formulae of Thai traditional medicine products containing cannabis as an active ingredient. The third category is made of folk medicines produced by folk healers (traditional medicines that are not yet listed by the national Thai traditional medicine formulary). These are currently under development and categorization. [13].

No study has specifically reported the use of medical cannabis in breast cancer survivors. However, the prevalence of cannabis use in Thailand increased from 2.2% in 2019 to 4.2% by 2021, following the legalization of cannabis for recreational use [14]. A study conducted in Northern Thailand showed that 40% of women with breast cancer had requested the use of medical cannabis from their physician, indicating an increasing interest in medical cannabis [15].

Moreover, a law was passed in 2019 which allowed the use of cannabis for medical purposes [14,16]. This law positioned cannabis on the national agenda and was amended in June 2022, allowing cannabis use for any purpose, including home cultivation and recreation [14]. However, the legalization of cannabis use has caused several problems in Thailand. First, the phrase “medical cannabis” remains confusing for Thais, who tend to use it without clear medical indications. They can purchase cannabis from several channels including illegal sources, home growers, traditional medicine providers, and modern medicine providers [16], making it more challenging to determine the safety of these products.

According to Kalayasiri and Boonthae [14], the prevalence of cannabis use in Thailand doubled after the legalization, overshadowing the use of illegal substances, such as kratom and methamphetamine. This is concerning since cannabis use can cause serious adverse effects in patients with breast cancer and reduce the therapeutic efficacy. In particular, the reduced therapeutic efficacy is evident in patients undergoing tamoxifen treatment [17,18].

Second, a person’s decision to use medical cannabis should rely on valid medical advice. However, owing to the lack of reliable resources, patients typically make an uninformed choice or rely on advice from their social circle, which can be misleading [16,19]. Further, people often seek cannabis-related information from easily accessible internet sources and social media platforms, rather than government-validated resources [16,19]. Previous studies on cannabis use, wherein data were collected from social networks, have faced skepticism due to a lack of rigorous scrutiny, allowing for potential bias or misinformation [19]. Moreover, the media has attempted to normalize cannabis use without a critical discussion of safety issues [20-22]. For instance, a content analysis study on cannabis-related information on Facebook in Thailand concluded that there was no discussion of the potential dangers of cannabis use [23].

The current trend of access to medical cannabis in Thailand is problematic since patients with breast cancer may access unvalidated information, which might be harmful, with reduced treatment efficacy. Furthermore, it appears unclear why people with breast cancer choose to seek medical cannabis–related content from internet sources and social media platforms. There is an urgent need to investigate the extent to which patients access cannabis-related information from internet sources and social media and evaluate the quality of the content available on these platforms.

This study aimed to (1) analyze the factors determining the quality of cannabis-related content for breast cancer care on internet sources and social media platforms and (2) examine the characteristics of cannabis-related content on internet sources and social media platforms accessed and used by Thai breast cancer survivors following cannabis legalization in 2019.

## Methods

### Study Design and Patient and Public Involvement Process

**Figure 1.**
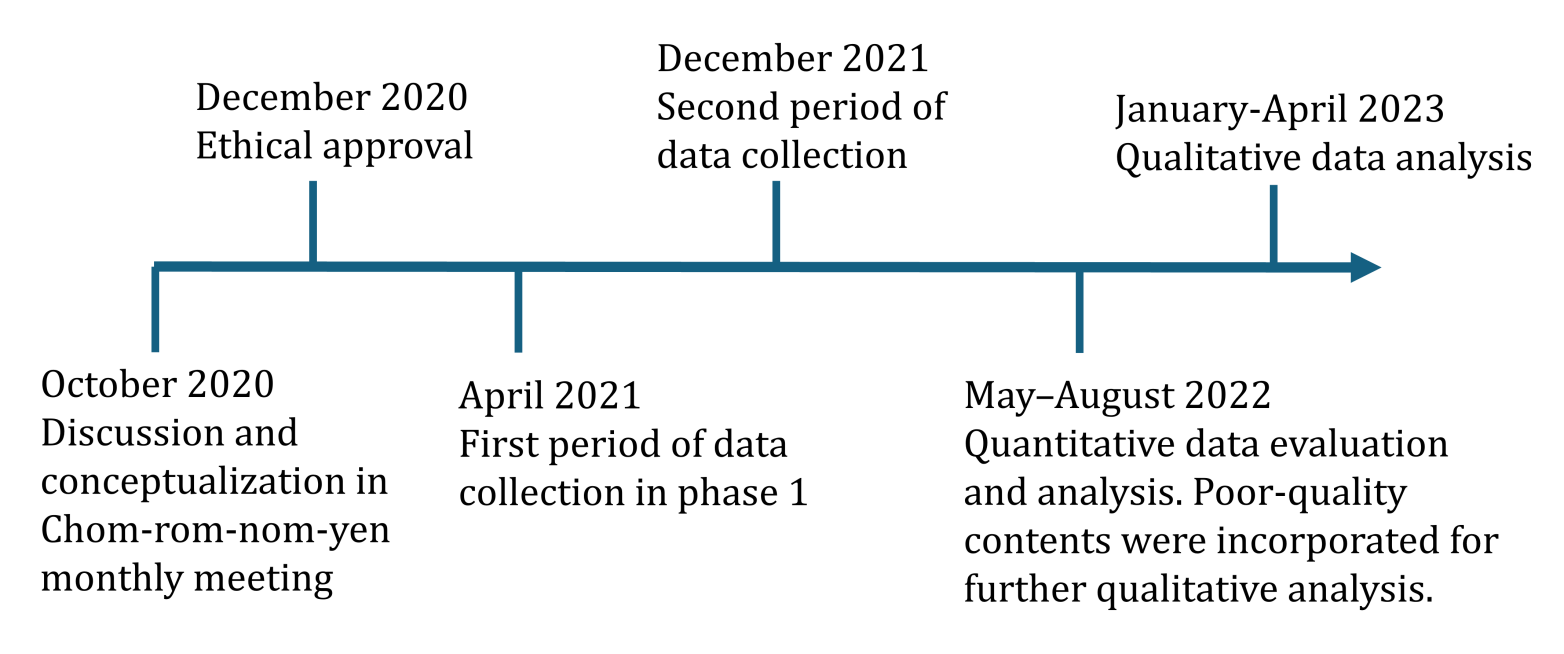
Study timeline.

This mixed methods sequential study was conducted in 2 phases between January 2021 and May 2022 at a tertiary hospital in Southern Thailand (Figure 1). We included members of the breast cancer support group “Chom-rom-nom-yen,” for patients and public involvement [24], to ensure representation of people with lived experiences of breast cancer. Chom-rom-nom-yen is a social enterprise of women living with breast cancer that aims to raise awareness, provide education, and advocate for and support women living with breast cancer. The organization was established in 2015 and currently comprises 397 breast cancer survivors and their caregivers from diverse socioeconomic backgrounds. The members were aged 30‐75 years, with the majority aged 50‐60 years. Most of the members resided in Southern Thailand.

The research began with a discussion of the initial ideas and the conceptualization of the problems and study design with the group in October 2020 at their in-person monthly meetings. Any group member could participate in the study as needed. A researcher (T Peerawong) joined the monthly meetings and discussed with Chom-rom-nom-yen members regarding the potential use of medical cannabis in Thai women with breast cancer postlegislation given that knowledge about medical cannabis use in breast cancer care in Thailand was limited at the time. The group was skeptical about the use of medical cannabis. However, some members indicated that they had already used cannabis-containing products, for example, cannabis tea and cannabis oil with no understanding of potential adverse and side effects. The group reached a consensus that using medical cannabis was worth further exploration. The subsequent group discussions via LINE, the most commonly used instant messaging platform in Thailand, further refined the focus of the study; 2 representatives agreed to join the study as core members to explore and evaluate the trustworthiness of medical cannabis–related information.

The subsequent activities were conducted and coordinated through Chom-rom-nom-yen; other members were contacted by a researcher (PS), the Chom-rom-nom-yen president. This direct involvement from the group helped ensure a focused and cohesive group participation.

The support group confirmed that they typically sought information from websites and popular social media platforms in Thailand namely Facebook, LINE, and YouTube.

### Ethical Considerations

The feedback from Chom-rom-nom-yen discussions was collated and included in the study design and ethical application. Ethical approval was obtained from the Institutional Review Board of the Faculty of Medicine, Prince of Songkla University (REC.63-531-7-1). Since the study involved the use of electronic and unidentifiable private information from internet sources and social media platforms, the ethical committee waived the requirement for informed consent [25].

The Institutional Review Board allowed the secondary analysis without additional consent. Pseudonyms were used to anonymize the content creators that were included in the data analysis process. Collected data were deidentified.

The data were securely stored in encrypted form in the Microsoft OneDrive storage of the university affiliation of the principal researcher, password-protected, and rendered compliant with the Thailand Personal Data Protection Act. Only the researchers (T Peerawong and T Phenwan) had access to the dataset. All the data will be deleted 5 years after the study completion, in compliance with the local regulations. Participants were not compensated.

### Procedure

Between April 2021 and December 2021, internet sources and social media content were gathered from women living with breast cancer who were members of the support group. Owing to the organic nature of Chom-rom-nom-yen, which acts as a social support group, demographic details of women participating in the research were not recorded, as anyone from the group could participate or opt out at any time.

The women were instructed to identify the information about medical cannabis that they found relevant from the available platforms during the study period (any websites, Facebook, LINE messages and posts, and YouTube). The unit of analysis included Facebook posts, blog posts, website articles, news updates, LINE messages, and videos. Participants then shared the URLs of these units of analysis with a researcher (NS) who collated all the data. This process was repeated twice over a 6-month period. The collected links were anonymized, merged, screened, and listed for further analysis by another researcher (T Peerawong).

### Quantitative Data Analysis

The first phase of the study involved quantitative content analysis and comprised a 3-part evaluation. The first part involved the collection of general information about the social network, such as its creators, platform type, content category, and upload date. The second part involved content quality analysis using the Quality Evaluation Scoring Tool (QUEST) and the Journal of the American Medical Association (JAMA) benchmarks. The QUEST is a validated tool that evaluates the quality of web-based health information. The instrument comprises 7 items: authorship, attribution, study type, conflicts of interest, currency, complementarity, and tone. Each item is scored on a scale of 0‐28 [26]. The JAMA benchmark is a 5-point scale that assesses: authorship, attribution, disclosure, and currency, with scores ranging from 0 to 4 [27]. Higher scores on both tools indicate higher quality. The third part of the evaluation involved expert assessment of several aspects of the content, such as the tone, accuracy, usefulness, and quality level, with scores ranging from 1 to 5.

The initial evaluation was performed by 2 research team members: one with more than 10 years of experience in radiation oncology and quality of life research (T Peerawong) and the other with 5 years of experience in social sciences research (SS). In this process, T Peerawong navigated the contents received from internet sources and social media platforms and discussed with SS until a consensus was reached. All selected content from internet sources and social media platforms was evaluated for content quality evaluation and expert analysis. All reviewers then independently followed the QUEST and JAMA evaluation guidelines. When necessary, questions were addressed to T Peerawong for clarification. The reviewers included a pharmacist specializing in herbal medicine with over a decade of experience (PP), a radiation oncologist (PT), and a general practitioner (NB), each with 3 years of experience. Additionally, a sixth-year medical student (PW) was included in the review team to provide variability in opinion.

Descriptive statistics were used to analyze the characteristics of social networks. Intraclass correlation was used to assess the interobserver reliability of the QUEST and JAMA benchmarks. The mean QUEST score among the 4 reviewers correlated with the expert opinion score for each aspect. A cut-off point for the QUEST score was determined by comparing QUEST scores with expert opinion scores, and a score of 3 or higher was considered to indicate high quality. After receiver-operating characteristic curve analysis, the Youden index was used to determine the optimal cut-off point owing to the simplicity of its calculation and interpretation [28]. Based on the QUEST scores, internet sources, and social media characteristics were categorized as high or poor content quality. Statistical significance was set at a *P* value <.05, and data analysis was conducted using R program (R Foundation for Statistical Computing, Vienna, Austria).

### Qualitative Data Analysis

#### Overview

The content deemed of poor quality in the first phase was further analyzed in the second phase of the study using Fairclough Critical Discourse Analysis approach [29]. The analysis aimed to identify visible or hidden discourses within the poor-quality datasets, as well as the ideologies, values, and assumptions of the content creators. The analysis was performed by T Phenwan, a family medicine doctor, and a qualitative researcher with expertise and research interests in breast cancer, quality of life research, and policy analysis. datasets were uploaded to Atlas.Ti to facilitate analysis. The researcher read or watched the content and analyzed the datasets using coding and memo features in Atlas.Ti. The analysis was conducted using the following 3 interrelated dimensions of critical discourse analysis.

#### Text Analysis (Description)

The microelements of the linguistic discourse in the datasets were examined. This included identifying the use of sensitizing words, linguistic devices (such as metaphors), choice of words, content structures, tones, and sentiments, and how discourses around the use of medical cannabis were framed.

#### Processing Analysis (Interpretation)

The situational context of the content was also explored, including how the content was produced and enabled the construction of new discourses or the sustainability of existing discourses. This involved examining content distributors and contributors, as well as considering who was included or excluded in content production, along with their positionalities towards medical cannabis.

#### Social Analysis (Explanation)

The socio-historical contexts and content producers were identified, along with overt and covert ideologies and hegemonies. The slant or style, intended audience, and intended purpose of the content creation were analyzed, along with the how and why of content structure, to explore how certain stakeholders or historical changes in cannabis-related policies influenced the created content.

The initial analysis was discussed among researchers (T Phenwan, MM [a sociologist specializing in social media analysis], and T Peerawong) via Microsoft Team meetings and email communications. The discussion continued until a consensus was reached.

## Results

### Quantitative Results

**Figure 2.**
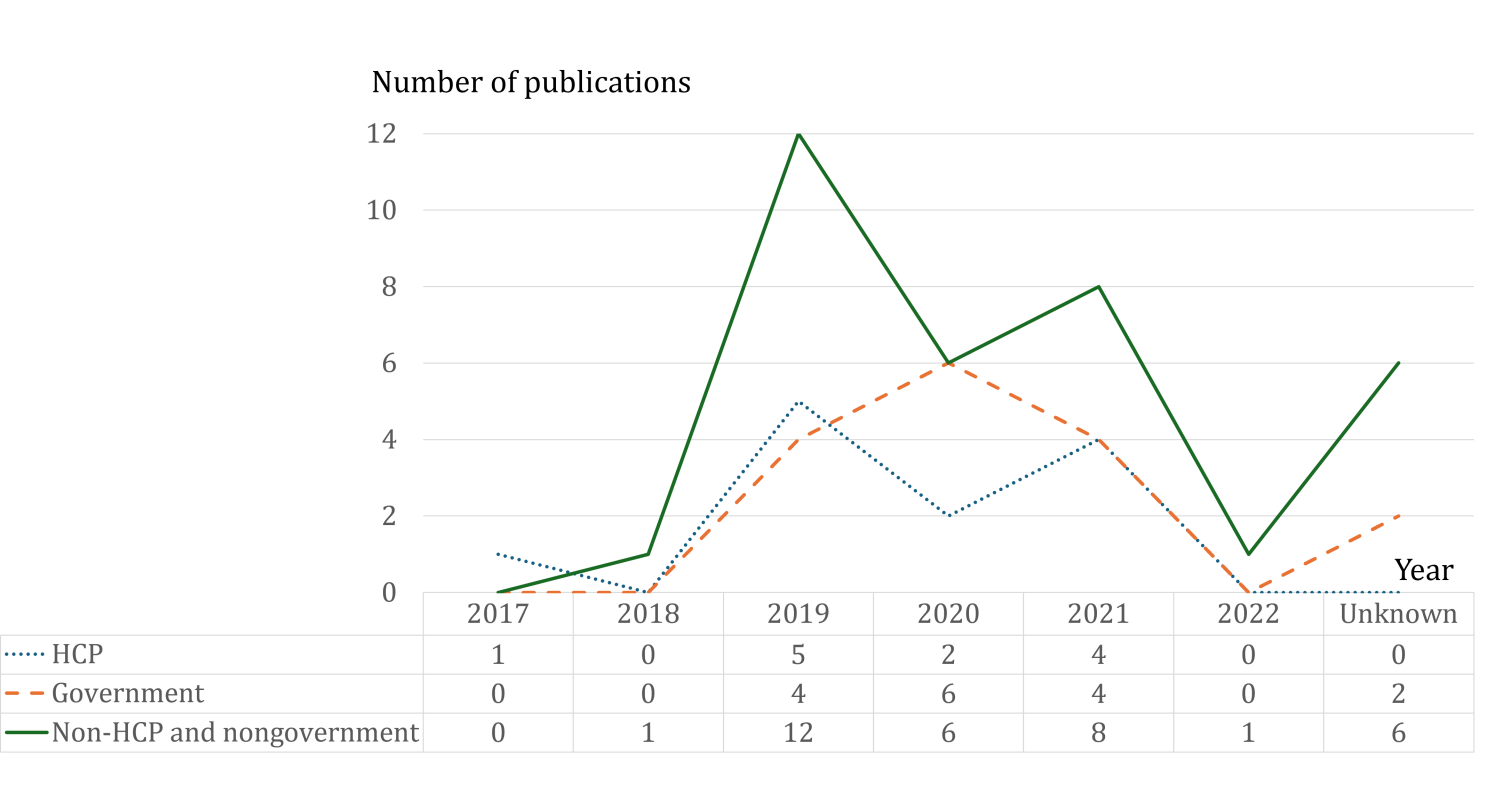
Distribution of content-uploading date categorized by content creators and years. HCP: health care professional.

Between April 2021 and December 2021, 62 internet content items were included and evaluated, all of which were in Thai. The most accessed content was created by news channels (21/62, 34%), followed by governmental sources (16/62, 26%), health care professionals (HCPs, 12/62, 19%), and alternative medicine providers (12/62, 19%). The most common internet sources and social media platforms were YouTube (30/62, 48%) and websites (19/62, 31%). Video content constituted 36 of the 62 content items (59%), with news (23/62, 37%) and advertisements (17/62, 27%) being the most frequent types (Table 1 and Figure 2).

Most of the contents were uploaded in 2019, with only 2 items uploaded prior to that year. These included news channel updates (sharing experience with cannabis) and a narrative review [30]. Most of the contents with unidentified uploading dates were created by alternative medicine providers. The URLs were sent to the reviewers, who individually evaluated the internet sources and social media content. Some internet sources and social media content were inaccessible and were not included during this stage. Four experts have reviewed, rated 62 websites, and provided 241 responses (Table 2). The evaluated content generally supported the use of cannabis, displaying fair information accuracy and usefulness. Only 64 of 241 expert opinion responses (26.5%) deemed the evaluated contents as high-quality.

The interobserver correlation of the QUEST score and JAMA benchmark between the 4 reviewers was 0.86 (*P*<.001) and 0.55 (*P*=.002), respectively. The QUEST score was 11.9 (SD 6), whereas the JAMA benchmark score was 2.5 (SD 1). The mean QUEST score was positively correlated with the mean JAMA benchmark score, as well as information accuracy, usefulness, and quality (Table 3). Additionally, the mean QUEST score showed a negative correlation with content tone. The correlation between the mean JAMA benchmark score and expert opinions was consistent with these findings.

For univariate analysis, a content quality score >3 based on expert opinion was considered indicative of high quality. To determine the optimal cut-off point of the content quality score, the receiver-operating characteristic curve was used to find a balance between sensitivity and specificity for binary classification. The area under the curve was 0.93, and the confidence interval ranged from 0.86 to 1.01. After applying the Youden index, a QUEST score of 15 was identified as the optimal cut-off point for differentiating between high and poor content quality. The sensitivity and specificity of this cut-off point were 81% and 98%, respectively. Univariate analysis of internet sources and social media characteristics revealed that content creator was the only variable with a significant difference between high and poor content quality, with high-quality content mostly originating from the government and HCPs (Table 4).

Twenty-eight contents were considered of poor quality and were mainly created by alternative medicine providers and news channels. These contents covered a range of topics, from instructions on how to plant cannabis to contraindications for medical cannabis use. Most contents (23/28, 82%) focused on the indications for cannabis use, instructions for medical cannabis use, and the general promotion of cannabis use. Only 1 news channel topic addressed the contraindications and side effects of medical cannabis. Of all contents from alternative medicine providers (9/11, 82%) were advertisements, with 8 out of 11 (73%) focusing on indications and instructions for cannabis use. No content from alternative medicine providers addressed the side effects or disadvantages of cannabis use. Of all content from news channels 11 out of 21 (52%) favored medical cannabis use. The information included the personal experiences of cannabis users, advertisements and discussions on cannabis cultivation, interviews with medical cannabis users, and interviews with HCPs. In terms of topics, 14 out of 21 (41%) of the contents were on indications and instructions regarding cannabis use, whereas 4 out of 21 (20%) were on pro-medical cannabis use.

**Table 1.** Baseline content characteristics (N=62).

Characteristic	Values, n (%)
Creators	
Alternative medicine provider	12 (19)
Government	16 (26)
Health care provider	12 (19)
Insurance company	1 (2)
Media (news channel)	21 (34)
Internet sources and social media platforms	
Facebook	12 (19)
YouTube	30 (48)
Website	19 (31)
Scientific journal	1 (2)
Content category	
Video	36 (58)
Text	20 (32)
Both	6 (10)
Content type	
News	23 (37)
Advertisement	17 (27)
How to grow cannabis	2 (3)
Academic article	2 (3)
General information on medical cannabis	5 (8)
Instruction of cannabis use	3 (5)
Experience of cannabis use	10 (16)

**Table 2.** Expert evaluation of internet and social media content (n=241).

Category	Values, n (%)
Support tone	190 (78.8)
Information accuracy	117 (48.5)
Usefulness	103 (42.7)
High quality	64 (26.5)

**Table 3.** Correlation of Journal of the American Medical Association (JAMA) benchmark and Quality Evaluation Scoring Tool (QUEST) score with expert opinion scores for tone, information accuracy, usefulness, and quality. Statistical analysis with Pearson correlation.

	JAMA	Quest	Tone	Information accuracy	Usefulness	Quality
JAMA	1	0.79^a^	−0.61^a^	0.77^a^	0.74^a^	0.82^a^
QUEST	0.79^a^	1	−0.87^a^	0.86^a^	0.90^a^	0.92^a^
Tone	−0.61^a^	−0.87^a^	1	−0.74^a^	−0.73^a^	−0.72^a^
Information accuracy	0.77^a^	0.86^a^	−0.74^a^	1	0.93^a^	0.91^a^
Usefulness	0.74^a^	0.92^a^	−0.73^a^	0.93^a^	1	0.93^a^
Quality	0.82^a^	0.92^a^	−0.72^a^	0.91^a^	0.93^a^	1

a *P*<.001.

**Table 4.** Univariate analysis of the Quality Evaluation Scoring Tool (QUEST) score between high and poor content.

		Poor, n (%)	High, n (%)	*P* value
Creators				<.001^a^
	Alternative medicine provider	11 (28)	0 (0)	
	Government	7 (17)	8 (50)	
	Health care provider	4 (10)	6 (38)	
	insurance company	1 (2)	0 (0)	
	Media (news channel)	17 (43)	2 (12)	
Internet sources and social media				.14^a^
	Facebook	8 (20)	3 (19)	
	Scientific journal	0 (0)	1 (6)	
	Website	10 (25)	7 (44)	
	YouTube	22 (55)	5 (31)	
Content category				.13^a^
	Both	5 (12)	0 (0)	
	Text	10 (25)	8 (50)	
	Video	25 (63)	8 (50)	

a Statistical analysis with Fisher exact test.

### Qualitative Results

All poor-quality contents were created between 2018 and 2021, with 5 contents having unspecified dates. Pseudonyms are used throughout this section to protect the identities of the content creators. Almost all content targeted members of the public; one content was explicitly created for policymakers and 4 contents were created for the public and HCPs.

Most content creators were anonymous, making it impossible to authenticate their identities. The creators almost always claimed undue credibility, including a cannabis merchant who claimed to have “five PhDs” and had “graduated from a university in the United States,” a farmer who was self-proclaimed as a “doctor,” or individuals who claimed to have had “cancer” that was cured by cannabis. The majority (21) of the poor-quality content portrayed a positive sentiment towards medical and general cannabis use. Seven contents had a neutral sentiment towards cannabis. Only 1 website created by the government superficially described the routes of administration of cannabis, with limited information regarding the contraindications and adverse effects of medical cannabis oil use (Multimedia Appendix 1).

Two main discourses were identified as follows: (1) advocacy for the normalization of cannabis use and (2) romanticization of cannabis as a panacea. The first discourse positions and normalizes cannabis as an everyday commodity for the public. In a YouTube video created by a university, a matter-of-fact detailed description of how to cultivate cannabis at home or in industrial-scale greenhouses is indicated by the following content:

*…There are 3 ways to cultivate cannabis. First, you can grow them outdoors. This method requires a minimal budget since we will be dependent on the natural sunlight as well as the weather. The drawback is that the crop is highly seasonal. There are also issues around insects and locusts…*

[Excerpt from a Facebook post from the Institute of Agricultural Study, Weed University, which was linked to YouTube]

The university also provided extensive information regarding cannabis cultivation on its Facebook page and website, further normalizing cannabis use for the public. Content from news outlets also empowered this discourse, since they tended to provide updates regarding cannabis, such as the impact of cannabis legislation at the time, newly opened cannabis clinics nationwide, or places to access free cannabis.

Great news! After [date], the geriatric clinic at temple A will provide free cannabis for 10,000 older people. 4,000 have already been enrolled so there are only 6,000 slots left. The registration slot is open twice a day so you should go there in the afternoon since the officers said it is not so packed. You have to be older than 65 to be eligible and it [cannabis oil] can cure your vision, hearing, tremors too!…[cannabis] is also free!

[Excerpt from “Cannabis clinic with new formula to be opened in 15 hospitals” on YouTube]

In the second discourse, all content creators used similar tones to persuade their target audiences, including the use of persuasive arguments with unsupported claims, obfuscations, or emotive statements. The content creators made claims to suit their underlying support for cannabis without providing appropriate evidence. For instance, one website claimed that cannabis oil can “completely cure 14 illnesses,” ranging from nausea and hemorrhoids to Alzheimer disease:

*…in terms of benefits of cannabis, abundant articles report various benefits of cannabis which includes […] Curing of asthma. All anti-asthmatic drugs are bad and have limitations due to their side effects [to your body], unlike cannabis which helps dilate your windpipe […] [cannabis] cures Parkinson’s and Alzheimer’s disease […] reducing the size of your hemorrhoid, once applied topically.*

[Excerpt from a blog post from “14 benefits of cannabis. Extremely useful! What can cannabis cure?”]

The content creator, an anonymous administrator, provided evidence supporting the claims; however, the source of the claims was not clarified.

Even content created by academic institutions used similar techniques to persuade the public to use medical cannabis, as seen in a YouTube video titled “Cannabis root helps with joint pain.”

*…[cannabis root] can help with your joint pain so well. Like, we still have limited use in the country, but it has been widely used overseas, like, by Persian doctors, Polish and English. They used cannabis root to help with joint pain, gouts, burns, like, everything really. Mix it with butter, all [of its use is] recorded. You can just boil the root and either apply it on your skin or even ingest it… [these benefits] are all evidence-based by modern medicine! And, like, it is super safe since it is, like, not addictive at all.*

[Excerpt from “Cannabis root helps with joint pain” on YouTube]

The next section discusses the findings from both phases of the study.

## Discussion

### Principal Findings

The study results align with the existing literature, revealing a significant shift in public sentiment regarding cannabis views and the use of internet sources and social media platforms. Prior to 2019, public sentiment from internet sources and social media platforms over cannabis use in Thailand was positive overall, with no discussion around its potential harm [23]. However, after 2019, there was a growing positive sentiment from both the public and patients towards medical cannabis use [15,31].

Research indicates that the legalization of medical cannabis use is linked to a reduced perception of its related risks and an increase in the frequency of cannabis use for both medical and recreational purposes [32]. In the United States, with the majority of cannabis legalization-related research, Melchior et al [33] concluded that the presence of medical cannabis–related laws and policies neither affects the first recreational use nor is correlated with reduced cannabis use, particularly among people younger than 25 years. However, the cannabis use frequency has increased among adults (aged at least 26 y) following its medical legalization [32,34]. Given that medical cannabis–related policies are nonhomogenous and subject to change, analyses that overlook heterogeneity in the key elements of these policies may inaccurately represent their effects on both recreational and medical cannabis use [34].

Furthermore, the legalization of medical cannabis use may reduce its associated stigma, thereby enhancing its acceptance and normalization among adults [35]. Additionally, the liberalization of cannabis-related laws has created new opportunities for individuals and businesses to promote their products, leading to a proliferation of advertisement and marketing efforts for cannabis-containing products, especially on the internet. This surge may be associated with increased cannabis use [36,37].

In Thailand, breast cancer survivors now have much greater access to various sources of cannabis-related information on internet sources and social media platforms. Notably, there has been a trend of survivors requesting more medical cannabis prescriptions over the years, despite a limited understanding of its potential adverse effects on their treatment [15].

This trend reflects a broader societal shift where individuals, including breast cancer survivors, increasingly rely on internet sources and social media platforms for health information [19,22,23,38]. The advantage of this information source is that the user has direct information access without geographical restrictions [38]. However, it is crucial to consider the quality and reliability of the health-related information available on internet. The lack of integration of information between mainstream health care and medical cannabis use may lead to adverse situations, particularly compounded by the often limited knowledge of cannabis use among both HCPs and medical cannabis users [39]. Moreover, web-based sources may vary in the quality of information provided, with some disseminating inaccurate or misleading information regarding medical cannabis use, a common pitfall associated with web-based health information [38].

As confirmed by the quantitative analysis, content creators are pivotal elements that can enable patients to accurately evaluate the veracity of web-based health information. This study identified an inverse relationship between the tonality of the content and its corresponding QUEST score, which is similar between medical cannabis use and glaucoma treatment [40]. That is, the content created by HCPs typically advises against the use of medical cannabis for glaucoma, which has superior quality over those created by non-HCPs. Jia et al [40] quantitatively analyzed the content quality and characteristics of popular internet search results related to glaucoma and medical cannabis use. The Sandvik score was used to quantitatively evaluate the content quality included in their study. This differs from our study because QUEST has been validated as an exclusive tool for mitigating web-based misinformation, demonstrating a significant positive correlation with the Sanvik scale. The QUEST incorporates additional evaluation criteria, including content tone, potential conflicts of interest, and extent of complementary information provided, thereby rendering the analysis more robust [26].

Our qualitative analysis generated 2 interrelated concerning discourses that underpin the poor-quality content accessed by breast cancer survivors. These 2 discourses validate, normalize, and somewhat romanticize the prevailing public perception of medical cannabis before its legalization in 2019 [14,23]. The first discourse tended to objectively update the public with information regarding cannabis use and cultivation in Thailand; nevertheless, it posed issues of concern as it did not discuss the potential adverse effects of cannabis. Instead, the first discourse implied that cannabis use should be normalized postlegislation, and its cultivation should be further encouraged.

This encouragement was compounded by the second discourse. The content creators used several persuasive techniques and false information to present cannabis as a “panacea” that cures or alleviates symptoms of various diseases, thereby downplaying its potential risks and side effects and contradicting its actual medical indications, such as intractable chronic pain, nausea, and vomiting [5]. Cannabis use is commonly perceived in internet sources and social media platforms as the last resort when modern medicine has failed in patient treatment or symptom alleviation [19,41]; nonetheless, this discourse repositions cannabis as the preferred therapy and is held in high esteem by all poor-quality content creators. This is alarming given these content creators claim to be “experts” in issues pertaining to cannabis and make false assertions without evidence.

This raises concerns about the potential impact of such narratives in shaping societal attitudes, particularly as they gain traction through their reproduction on internet sources and social media platforms. However, the quality and credibility of the information disseminated through these channels exacerbate this concern.

The influence of these discourses on fostering a more accepting stance towards medical cannabis use in the public is disconcerting, particularly because of the lack of discussion on the potential adverse effects of cannabis on cancer treatment. This information gap poses a considerable risk, as patients with breast cancer and the general public can access content created by anyone on the internet. While this accessibility empowers individuals, it also introduces the possibility that people may accidentally hurt themselves through uninformed cannabis use. Safeguarding the well-being of those seeking guidance and insight requires addressing this misinformation and ensuring that individuals are equipped with the knowledge needed to make informed decisions regarding cannabis use [36,42], particularly in the context of cancer treatment.

In Thailand, individuals who create and share such content face no legal repercussions because cannabis use is legal for medical and recreational purposes [16]. Findings from this study suggest that the Thai Government needs to address the varying cannabis-related content on the internet. This could be achieved by implementing stricter advertising and marketing policies for cannabis promoted through web-based sources and social media platforms or establishing mechanisms for accountability [37]. This approach could help protect the public and those living with breast cancer who may be more influenced by misleading or inaccurate information about cannabis products.

To our knowledge, this is the first mixed method study to analyze the quality of cannabis-related internet sources and social media content in Thailand. Although not all the findings are generalizable, certain aspects could be transferred to other contexts [43]. Specifically, we discussed the varying quality of medical cannabis–related internet content that requires mitigation and the need to regulate such content, which are more applicable to this field of study.

The integrated results from both study phases provide more contextual insights into the medical cannabis–related contents shared over the internet, and the underlying postlegislation discourses in Thailand. To this end, our interdisciplinary team members, including one person with lived experiences, offer a more comprehensive understanding and insight.

Moreover, this is the first study in Thailand to include people with lived experiences of breast cancer as research partners throughout the project. Preliminary results from this study have been shared with the breast cancer support group via short lay summaries and group representatives.

### Future Implications

One way to tackle the internet flood of biased information on cannabis is to promote health literacy. Health literacy refers to “the personal characteristics and social resources needed for individuals and communities to access, understand, appraise, and use information and services to make decisions about health” [44]. Health literacy has a direct correlation with better health outcomes and health-seeking information behavior [45,46]; hence, it should be promoted. However, approximately half of the Global Southern population, including the Thai population, have inadequate health literacy levels. Low health literacy levels are more prevalent among people from rural areas and those with low educational levels [45]. This highlights the importance of addressing health literacy divides, particularly in regions with limited access to health care resources and educational opportunities.

Another relevant aspect to consider is the notion of electronic health literacy, which is particularly significant in today’s society where individuals have easy access to vast amounts of information via the internet [47-49]. Therefore, it is imperative to promote public health literacy and digital health literacy to ensure that people, including patients with breast cancer, can critically evaluate the quality and validity of any content they access [50]. This can be achieved through several strategies, such as addressing the unmet needs of patients with breast cancer or developing easily accessible and user-friendly educational materials [50,51]. These recommendations are equally applicable to medical cannabis–related content.

As revealed by this study and supported by existing literature, there is a notable scarcity of validated educational resources on medical cannabis [52]. This deficiency poses a challenge as HCPs may lack the necessary knowledge to offer well-informed advice to their patients. Conversely, patients driven by a growing interest in medical cannabis use may resort to unvalidated sources, exposing themselves to inaccurate information. To mitigate this, the government should establish a dedicated task force that would prioritize the development of validated educational resources for both HCPs and the public. Future research should also focus on benchmarking and validating cannabis-related content for HCPs and end users.

In Thai contexts, most patients still rely on HCPs for health-related information [45,46]. Therefore, it may be beneficial to begin with educational resources specifically tailored to HCPs. This strategic approach recognizes the pivotal role that HCPs play in disseminating accurate information and ensures that they are well-equipped with the necessary knowledge about medical cannabis.

It is noteworthy to further explore the unmet needs and expectations of patients that drive them to seek out additional information from the internet. This could be explored using qualitative research designs, which would allow for a deeper understanding of patient’s needs and expectations, thereby improving the ability of HCPs to meet their informational needs more effectively.

Finally, although the study predominantly focused on identifying “poor-quality” content, it may lack an in-depth exploration of sources or characteristics associated with high-quality content. This limits a comprehensive understanding of the factors contributing to reliable medical cannabis–related information. Therefore, “high-quality” content should be further explored in the future.

### Limitations

This study has some limitations. First, the analyzed contents were exclusively obtained from a single group of women living with breast cancer. The contents may not fully represent all publicly available cannabis-related content in Thailand. However, the results offer valuable insights into the current state of publicly available information on cannabis in the country. Future studies should explore a broader range of cannabis-related sources available to the public, as well as patients, to provide a fuller picture of the situation. Additionally, future research should encompass a more diverse range of sources and larger sample sizes to ensure a more comprehensive analysis of cannabis-related content.

Second, the study faced challenges in determining content quality due to accessibility issues. Some experts could not access certain content pages (n=6), albeit the reasons for these difficulties were not documented by participants from Chom-rom-nom-yen. This barrier potentially compromised the thoroughness of our evaluation, raising concerns regarding the accuracy and completeness of our content quality analysis. This emphasizes the critical need to ensure that all pertinent contents are readily accessible to researchers and experts for effective and comprehensive evaluation.

In the qualitative analytical phase, the study specifically focused on Thai cannabis-related content shared on internet sources and social media platforms. Therefore, the results may not be fully generalizable to other populations with different cultural backgrounds, health care systems, or attitudes towards cannabis. This study primarily analyzed content quality based on the creator’s identity. However, it may have overlooked other factors, such as audience engagement, context, and evolving social media trends, which could have influenced the perception of content quality. The categorization of contents as “high” or “poor” may have been subjective and influenced by the perspectives of the project members. Different individuals or groups may have varying opinions on what constitutes reliable and accurate information regarding medical cannabis. Furthermore, internet sources and social media content are highly dynamic, and this study may not have captured the evolving nature of information over time.

### Conclusions

This study highlights the varying quality of medical cannabis–related information accessed and shared on internet sources and social media platforms among Thai breast cancer survivors. Given that content creators are the primary predictors of high content quality, future studies should explore a broader range of cannabis-related sources available to the public and patients for a more comprehensive understanding of the situation.

## Supplementary material

10.2196/55300 Multimedia Appendix 1 Bad content characteristics and identified discourses.

## Abbreviations

HCP: health care professional
JAMA: Journal of the American Medical Association
QUEST: Quality Evaluation Scoring Tool
