# Supplementary material for: Evaluating Online Cannabis Health Information for Thai Breast Cancer Survivors Using the Quality Evaluation Scoring Tool (QUEST): Mixed Method Study
Source: JMIR Cancer. 2024 Dec 24;10:e55300. doi: 10.2196/55300 (PMC11693783; doi:10.2196/55300)
Supplement: Multimedia Appendix 1 [file cancer-v10-e55300-s001.docx]

| Bad content characteristics and identified discourses | | | | | | | |
| --- | --- | --- | --- | --- | --- | --- | --- |
| Platform | Date created | Title  (pseudonyms) | Content creator | Sentiment | Content tone | Target audiences | Discourses identified |
| **Facebook** | August 2018 | Uncle Tam | Alleged patient who had “unspecified cancer” | Positive | Persuasive, informal, and emotive with purposeful obfuscation and false claims of evidence | Members of the public | Romanticization of cannabis as a panacea |
|  | October 2018 | Weed Wizard | Alleged alternative “medicine professional” with “five PhDs” | Positive | Persuasive, informal, and emotive with purposeful obfuscation and false claims of evidence | Members of the public | Romanticization of cannabis as a panacea |
|  | Unspecified | The Institute of Agricultural Study, Weed University | Higher education institute | Positive | Persuasive, informative, and formal | Members of the public | Advocacy for the normalization of cannabis use |
|  | January 2021 | The Institute of Agricultural Study, Weed University | Higher education institute | Neutral | Informative | Members of the public | Advocacy for the normalization of cannabis use |
|  | May 2021 | How to grow your cannabis outdoor - special blend | Anonymous Facebook content creator | Neutral | Informative | Members of the public | Advocacy for the normalization of cannabis use |
|  | May 2019 | Weed for all United, Thailand - Southern blend | Anonymous Facebook content creator | Positive | Persuasive, informative, and formal |  | Romanticization of cannabis as a panacea |
|  | October 2020 | Recording of medical cannabis seminar | Higher education institute | Positive | Persuasive, informative, and formal | -Members of the public  -Academia | Advocacy for the normalization of cannabis use |
|  | January 2021 | Cannabis Episode 1: The largest cultivation plant in the Association of Southeast Asian Nations | Higher education institute | Positive | Persuasive, informative, and formal | -Members of the public  -Academia | Advocacy for the normalization of cannabis use |
| **Website** | Unspecified | My experience of extracting cannabis oil | Academia who worked at a hospital | Neutral | Academic and formal | Policymakers and healthcare professionals | Advocacy for the normalization of cannabis use |
|  | November 2020 | 14 benefits of cannabis. Extremely useful! What can cannabis cure? | Anonymous website administrator | Positive | Journalistic, informative, and reassuring with false claims of evidence | -Members of the public | Romanticization of cannabis as a panacea |
|  | April 2019 | Exclusive from Uni “X”. Cannabis can cure cancer in rats. Shall we try it in humans too? | News outlet | Positive | Journalistic and supportive | -Members of the public | Advocacy for the normalization of cannabis use |
|  | Unspecified | Cannabis oil: Dr Dhesi formula | Division of Alternative Medicine, Ministry of Public Health, Thailand | Neutral | Directive and formal | -Members of the public  -Healthcare professionals | Advocacy for the normalization of cannabis use |
|  | Unspecified | How cannabis cures cancer | Private clinic run by medical doctors | Positive | Persuasive, informative, and formal | -Members of the public  -Healthcare professionals | Romanticization of cannabis as a panacea |
|  | Unspecified | My one year of living with terminal cancer and cannabis | Private clinic run by medical doctors | Positive | Persuasive, informative, and formal | -Members of the public  -Healthcare professionals | Romanticization of cannabis as a panacea |
|  | May 2019 | Magic! Cannabis oil kills cancer! Retired teacher with stage 4 cancer can walk again! | News outlet | Positive | Emotive, hyperbolic, and informal | -Members of the public | Romanticization of cannabis as a panacea |
| **YouTube** | May 2019 | Side effects of cannabis from different administration routes | Medical doctor | Neutral | Informative | -Members of the public | Advocacy for the normalization of cannabis use |
|  | June 2020 | CBD for all - curing your stress, insomnia, depression, and psychosis | Alleged alternative medicine professional with 'five PhDs' | Positive | Persuasive and emotive with purposeful obfuscation and false claims without evidence | -Members of the public | Romanticization of cannabis as a panacea |
|  | August 2019 | New product to cure your pain with CBD oil | Private company | Positive | Persuasive and emotive with false information and unsupported claims | -Members of the public | Romanticization of cannabis as a panacea |
|  | August 2019 | Cannabis clinic with new formula to be opened in 15 hospitals | News outlet | Positive | Informative and persuasive with some evidence to support claims | -Members of the public | Romanticization of cannabis as a panacea |
|  | December 2019 | Coffee talk: why can’t patients have access to cannabis? | News channel interview with doctors | Positive | Persuasive and emotive with false information and unsupported claims | -Members of the public | Romanticization of cannabis as a panacea |
|  | April 2021 | Cannabis for health | News channel interview with a pharmacist | Neutral | Informative | -Members of the public | Advocacy for the normalization of cannabis use |
|  | October 2020 | Where you can get free weed | Presenter from a private company | Positive | Informative | -Members of the public | Advocacy for the normalization of cannabis use |
|  | April 2021 | How to grow weed for dummies: Outdoor, Greenhouse, and Indoor | Alleged 'medical doctor' | Neutral | Informative | -Members of the public | Advocacy for the normalization of cannabis use |
|  | April 2021 | Weed formula in Thai traditional medicine | Alternative medicine provider | Positive | Informative | -Members of the public | Romanticization of cannabis as a panacea |
|  | April 2021 | How to use cannabis to cure joint pain | Medical doctor | Positive | Informative and persuasive with some evidence to support claims | -Members of the public | Romanticization of cannabis as a panacea |
|  | Nov 2018 | Cannabis can cure cancer? Or not? | News outlet with “cancer” patients | Positive | Persuasive and emotive with false information and unsupported claims | -Members of the public | Romanticization of cannabis as a panacea |
|  | August 2019 | Minister of Public Health orders 1 million bottles of Dr Dhevi formula CBD oil | News outlet | Positive | Informative | -Members of the public | Advocacy for the normalization of cannabis use |
|  | December 2020 | Cannabis root helps with joint pain | Alternative medicine institute | Positive | Persuasive and emotive with false information and unsupported claims | -Members of the public | Romanticization of cannabis as a panacea |
|  | May 2021 | Insomnia: my experience of tackling insomnia with cannabis | Alternative medicine private clinic. An interview with a patient | Positive | Persuasive and emotive with false information and unsupported claims | -Members of the public | Romanticization of cannabis as a panacea |
|  | July 2019 | Can cannabis actually cure insomnia? Is it available in Thailand | Faculty of Pharmacy of a university. Interview with the public | Positive | Informative | -Members of the public | Romanticization of cannabis as a panacea |
|  | February 2021 | Cannabis: the new super plant for the post-COVID-19 era! | Medical doctors | Positive | Persuasive and emotive with false information and unsupported claims | -Members of the public | Romanticization of cannabis as a panacea |
|  | December 2020 | How to use cannabis leaf safely | Researcher from an institute | Positive | Persuasive and emotive with false information and unsupported claims | -Members of the public | Romanticization of cannabis as a panacea |
|  | July 2019 | Dhevi oil. Cannabis oil for patients: an agent of change | Interview with a farmer who was self-proclaimed as a “doctor” | Positive | Persuasive and emotive with false information and unsupported claims | -Members of the public | Romanticization of cannabis as a panacea |
